# Triggered radiosensitizer delivery using thermosensitive liposomes and hyperthermia improves efficacy of radiotherapy: An *in vitro* proof of concept study

**DOI:** 10.1371/journal.pone.0204063

**Published:** 2018-09-18

**Authors:** Helena C. Besse, Clemens Bos, Maurice M. J. M. Zandvliet, Kim van der Wurff-Jacobs, Chrit T. W. Moonen, Roel Deckers

**Affiliations:** 1 Center of Imaging Sciences, University Medical Center Utrecht, Utrecht, the Netherlands; 2 Department of Clinical Sciences of Companion Animals, Faculty of Veterinary Medicine, Utrecht University, Utrecht, the Netherlands; University of South Alabama Mitchell Cancer Institute, UNITED STATES

## Abstract

**Introduction:**

To increase the efficacy of chemoradiation and decrease its toxicity in normal tissue, a new concept is proposed, local radiosensitizer delivery, which combines triggered release of a radiosensitizer from thermosensitive liposomes with local hyperthermia and radiotherapy. Here, key aspects of this concept were investigated *in vitro* I) the effect of hyperthermia on the enhancement of radiotherapy by ThermoDox (thermosensitive liposome containing doxorubicin), II) the concentration dependence of the radiosensitizing effect of doxorubicin and III) the sequence of doxorubicin, hyperthermia and radiotherapy maximizing the radiosensitizing effect.

**Methods:**

Survival of HT1080 (human fibrosarcoma) cells was measured after exposure to ThermoDox or doxorubicin for 60 minutes, at 37 or 43°C, with or without irradiation. Furthermore, cell survival was measured for cells exposed to different doxorubicin concentrations and radiation doses. Finally, cell survival was measured after applying doxorubicin and/or hyperthermia before or after irradiation. Cell survival was measured by clonogenic assay. In addition, DNA damage was assessed by γH2AX staining.

**Results:**

Exposure of cells to doxorubicin at 37°C resulted in cell death, but exposure to ThermoDox at 37°C did not. In contrast, ThermoDox and doxorubicin at 43°C resulted in similar cytotoxicity, and in combination with irradiation caused a similar enhancement of cell kill due to radiation. Doxorubicin enhanced the radiation effect in a small, but significant, concentration-dependent manner. Hyperthermia showed the strongest enhancement of radiation effect when applied after irradiation. In contrast, doxorubicin enhanced radiation effect only when applied before irradiation. Concurrent doxorubicin and hyperthermia immediately before or after irradiation showed equal enhancement of radiation effect.

**Conclusion:**

*In vitro*, ThermoDox resulted in cytotoxicity and enhancement of irradiation effect only in combination with hyperthermia. Therefore hyperthermia-triggered radiosensitizer release from thermosensitive liposomes may ultimately serve to limit toxicities due to the radiosensitizer in unheated normal tissue and result in enhanced efficacy in the heated tumor.

## Introduction

Radiotherapy (RT) is often used in the treatment of solid tumors, either as monotherapy or in combination with another treatment modality such as chemotherapy or surgery [1]. For many tumors RT makes a significant contribution to a successful treatment, although it is also accompanied by a serious risk for undesired damage of normal tissue in the beam path and surrounding the tumor. These side effects limit the radiation dose that can be given to the tumor, which could lead to suboptimal radiotherapy treatment [2]. This and intrinsic radioresistance of some tumors contributes to the high recurrence rate observed for several cancers [3]. One of the approaches to increase the local efficacy of RT is to combine it with chemotherapy, i.e. chemoradiation [4].

Administrating chemotherapy and RT simultaneously, i.e. concurrent chemoradiation, is currently part of the standard of care for many difficult to treat cancers, including gastric, head and neck and cervical cancers and sarcomas [5–8]. While concomitant chemoradiation has improved disease control as well as survival, it also leads to significantly higher toxicities compared to sequential treatment [9] or RT as a single treatment [10].

To reduce the toxicity of chemotherapy, a common approach is to encapsulate these agents in nanomedicine, e.g. liposomes [11, 12]. In addition, nanomedicine formulations lead to tumor specific drug accumulation due to the enhanced permeability and retention (EPR) effect. The EPR effect is only present in the tumor and is caused by discontinuous endothelial lining and lack of efficient lymphatic drainage, resulting in preferential accumulation of liposomes in the tumor [13]. In animal models the EPR effect leads to increased efficacy of liposomal doxorubicin compared to free doxorubicin (DOX) [14]. In patients with metastatic breast cancer, liposomal doxorubicin reduces the toxicity, while maintaining equal efficacy [11]. The lack of improvement in progression free survival and overall survival of patients treated with liposomal doxorubicin is attributed to heterogeneity of the EPR effect in human tumors [15]. Furthermore, it is argued that the slow DOX release from liposomes, 50% drug release in 118 hours [16], leads to limited bioavailability of the drug in the tumor [16, 17].

To improve the bioavailability of chemotherapeutic agents, trigger-sensitive nanosystems combined with a local trigger are proposed in literature [18], for example thermosensitive liposomes (TSL) in combination with heat as a local trigger [19]. Combining TSL with local hyperthermia (HT) (i.e. 40–42°C) results in fast drug release, 60% drug release in 20 seconds at a temperature of 41.3°C [20]. This fast release is caused by the increase in permeability of the phospholipid bilayer of the liposomes at the phase transition temperature (~41.3°C) [21]. Several preclinical studies show that this approach leads to increased tumor drug concentration, to a more homogeneous drug distribution in the tumor [19, 22, 23] and to increased survival compared to free drug *in vivo* [24, 25].

Here, a new concept, local radiosensitizer delivery, is proposed in which radiosensitizers are released from the TSL by locally applying hyperthermia to the tumor in combination with conventional radiotherapy. The overall aim of this concept is to reduce the toxicity of the radiosensitizer in normal tissue. In addition, the increased radiosensitizer concentration in the tumor could lead to stronger radiosensitization effect.

The concept of local radiosensitizer delivery implies a combination of three treatment modalities, i.e. radiosensitizer, HT and RT. Within this concept HT is, besides a trigger for radiosensitizer release, also a known chemosensitizer [26, 27] and radiosensitizer [28, 29]. Consequently, the sequence of applying the three treatment modalities will largely influence the overall effect.

In this study we investigated *in vitro* several important aspects of the triggered radiosensitizer delivery concept. Our objectives were to investigate I) the effect of HT on the enhancement of RT by TSL loaded radiosensitizer, II) the concentration dependent radiosensitizing effect of DOX and III) the sequence of DOX, HT and RT maximizing the radiosensitizing effect. For this purpose ThermoDox, a TSL containing DOX, is used to achieve triggered radiosensitizer delivery, since it is already available for clinical trials. DOX is in this concept used as a radiosensitizer, although in the clinic it is often used as a chemotherapeutic agent. We verified the cell survival of cells treated with ThermoDox in the presence and absence of HT with and without RT. For the concept of triggered radiosensitizer delivery, in the absence of HT ThermoDox should not affect the cell survival nor lead to radiosensitization, whereas in the presence of HT ThermoDox should affect the cell survival and lead to radiosensitization comparable to DOX. Subsequently, DOX concentration dependent enhancement of the RT was investigated. For this concept an increase in radiosensitizer would preferably result in an increase in radiosensitization. Finally, the optimal sequence of the modalities DOX, HT and RT was determined to maximize the radiosensitization.

## Materials and methods

### Cell culture

HT1080 (human fibrosarcoma) cells, obtained from ATCC (ATCC number CCL-121), were cultured in Minimum Essential Medium (MEM) (Gibco) supplemented with 292 mg/L L-glutamine (Sigma), 110 mg/L sodium pyruvate (Sigma) and 10% Fetal Bovine Serum (Sigma F7524). During all treatments 10 mM HEPES (Sigma) was supplemented to the medium to stabilize the pH. Cells were cultured at 37°C in 5% CO_2_ in an air humidified incubator and were regularly tested for mycoplasma contamination.

### Treatment modalities

Hyperthermia, in the range of 37 to 43°C, was performed by incubating cells in a water bath (WNE14l, Memmert, Schwabach, Germany). The bottom of the plate was in direct contact with water inside the water bath, S1 Fig. This water bath setup was validated using a calibrated fiber optic temperature probe (Neoptix Reflex, Neoptix, Cancada, LP) showing that cell medium reached its target temperature within 10 minutes after being positioned in the water bath.

Chemotherapy treatment was performed by incubating cells for 1 hour with doxorubicin·HCl (DOX) (Guanyu Biotechnology Co., LTD, Xi’an, China) or ThermoDox (Celsion Corporation, Lawrenceville, NJ, USA). Stock solution of DOX was prepared at a concentration of 5 mg/mL and ThermoDox was obtained at a DOX concentration of 2 mg/mL. Before each treatment, DOX and ThermoDox solutions were freshly prepared from stock solution in the concentration range of 0 to 0.24 μg/mL.

Radiotherapy was performed by a single dose of X-ray by a linear accelerator (Elekta Precise Linear Accelerator 11F49, Elekta, Crawley, United Kingdom, 6-MV) in the range of 0 to 8 Gy. During the irradiation cells were positioned on top of 2 cm polystyrene and submerged in a 37°C water bath, S1 Fig. By applying the radiation from below, the polystyrene ensures proper dose build-up, while the water ensures backscatter to occur. Due to logistics, a time interval of 45 minutes was present between DOX and/or HT treatment and RT.

### Cell survival assay

Cell survival was measured by clonogenic assay, according to Franken et al. [30]. Briefly, at day -1 between 150 and 20,000 cells, depending on the treatment, were seeded in triplicate in 6-well plates and incubated under regular culture conditions. At day 0 cells were treated and subsequently incubated under regular culture conditions. At day 7 cells were fixed and stained with 6% glutaraldehyde and 0.05% crystal violet. Colonies of at least 50 cells were counted. The survival fraction was calculated either relative to untreated samples (0 μg/ml DOX, 37°C and 0 Gy) or relative to samples treated with corresponding treatment of DOX and/or HT at a RT dose of 0 Gy. Representative clonogenic assay images and its analysis are included in S2 Fig.

### Double strand breaks measurement by flow cytometry of γH2AX staining

Cells were harvested by trypsinization, fixed in 1% PFA and permeabilized in 70% ethanol overnight at -20°C. Subsequently, non-specific antibody binding was blocked by incubating cells for 5 minutes with 1% BSA/0.2% Triton X-100/PBS at room temperature. Staining of γH2AX was performed by incubating cells for 30 minutes with FITC-labeled anti-H2AX mouse IgG1 antibody (1:20; #613403, Biolegend, London, United Kingdom) at room temperature. Next, cells were washed and 5 μg/ml propidium iodine (Sigma-Aldrich) and 100 μg/ml RNase (ThermoFisher) in PBS was added to the samples to normalize the γH2AX signal for the DNA concentration. Finally, samples were measured by flow cytometry (BD FACSCanto II) and analyzed by FlowJo software (FlowJo, Ashland, OR). The percentage γH2AX positive cells was normalized to untreated cells (0 μg/ml DOX, 0 Gy at 37°C). Representative flow cytometry images and its analysis are included in S3 Fig.

### The effect of ThermoDox and DOX in combination with HT and RT on cell survival

Cells were exposed to ThermoDox (0 or 0.02 μg/ml) or DOX (0 or 0.02 μg/ml) for 1 hour at 37 or 43°C and subsequently irradiated with different radiation doses (0 to 8 Gy). Finally, cell survival was determined as described above.

### The radiosensitizing effect of DOX

Cells were exposed to DOX at different concentrations (0, 0.01, 0.02 or 0.06 μg/ml) for 1 hour at 37°C and subsequently irradiated with different radiation doses (0 to 8 Gy). Finally, cell survival was determined as described above.

### The effect of the combination of individual treatment modalities on cell survival

For the combination of concurrent DOX and HT, cells were exposed to DOX concentrations in the range of 0 to 0.24 μg/ml for 1 hour at 37, 42 or 43°C. For the combination treatments of RT and DOX and/or HT, cells were exposed to DOX (0 or 0.02 μg/ml) for 1 hour at 37, 42 or 43°C before or after irradiation with different radiation doses (0 to 8 Gy). Finally, cell survival was determined as described above.

### The effect of DOX with and without HT on DNA damage

Cells were exposed to DOX (0, 0.02 or 0.06 μg/ml) for 1 hour at 37 or 43°C. Afterwards, cells were incubated under regular culturing conditions as described above. At time points 2 and 26 hours after DOX removal DNA damage was measured by flow cytometry of γH2AX staining as described above.

### The effect of the combination of individual treatment modalities on DNA damage

Cells were exposed to DOX (0 or 0.02 μg/ml) for 1 hour at 37 or 43°C, subsequently cells were irradiated with 0 or 4 Gy. Afterwards, cells were incubated under regular culturing conditions as described above. At time points 45 minutes and 24 hours after irradiation, DNA damage was measured by flow cytometry of γH2AX staining as described above.

### Statistical analysis

All data is presented as mean, with error bars representing the standard deviation based on a minimum of 3 independent experiments. Cell survival data by clonogenic assay were statistically tested in SPSS 23 (IBM, Armonk, NY, USA) by linear regression as described by Franken et al [30]. DSB measurements by γH2AX were statistically tested in GraphPad Prism 7 (GraphPad Software, Inc, San Diego, CA) by comparison of groups by a paired t-test. Differences between groups with p < 0.05 were considered statistically significant.

## Results

### The effect of triggered release of DOX from a TSL in combination with RT

**Fig 1:**
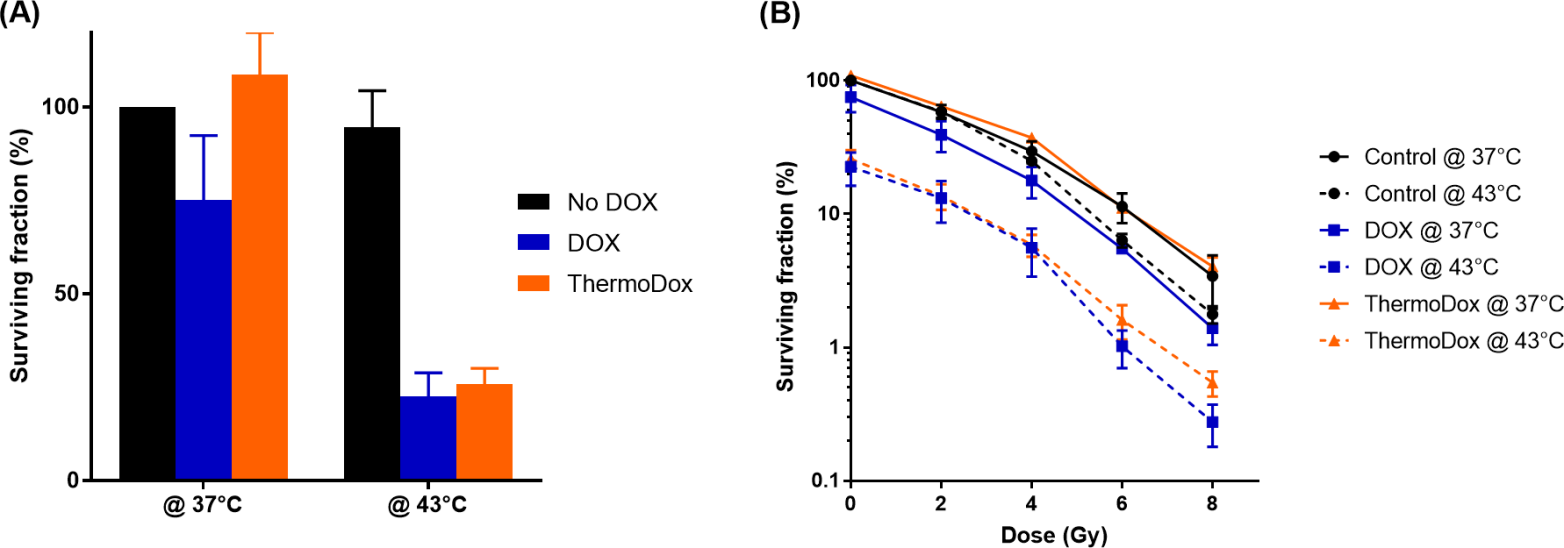
Cytotoxicity of free DOX and DOX from ThermoDox at different temperatures with and without RT. Surviving fraction of HT1080 cells exposed to DOX (0.02 μg/mL) or ThermoDox (0.02 μg/mL) for 1 hour at 37°C or 43°C without (A) or with (B) RT. ThermoDox at 37°C, with or without RT, resulted in equal toxicity compared to treatment without a drug, however ThermoDox at 43°C resulted in comparable toxicity compared to DOX treatment.

First the cytotoxicity of ThermoDox at different temperatures was investigated. Fig 1A shows the surviving fraction of cells incubated with DOX or ThermoDox at a concentration of 0.02 μg/ml for 1 hour at 37 or 43°C, relative to the untreated samples (0 μg/ml DOX at 37°C). Cells treated with ThermoDox at 37°C showed similar survival compared to untreated cells (0 μg/ml DOX). In contrast, cells treated with ThermoDox at 43°C showed a similar surviving fraction compared to cells exposed to free DOX at 43°C, which is caused by the fast DOX release from the TSL at elevated temperatures. Incubation of cells at 43°C by itself resulted in limited toxicity, a surviving fraction of 94.46% ± 9.92% was obtained. Note that these cells are rather heat resistant compared to other cell lines [31, 32], S4 Fig.

Subsequently, the cytotoxicity of the combination of ThermoDox and RT was investigated at different temperatures. Fig 1B shows surviving fraction as function of radiation dose for cells incubated for 1 hour with DOX or ThermoDox at 37 or 43°C followed by RT treatment. The surviving fraction is presented relative to untreated sample (0 μg/ml DOX, 37°C, 0 Gy). As a function of RT dose, ThermoDox at 37°C resulted in similar survival as RT as a single treatment, whereas ThermoDox at 43°C resulted in comparable toxicity as DOX at 43°C followed by RT.

### Concentration dependent radiosensitization effect of DOX

**Fig 2:**
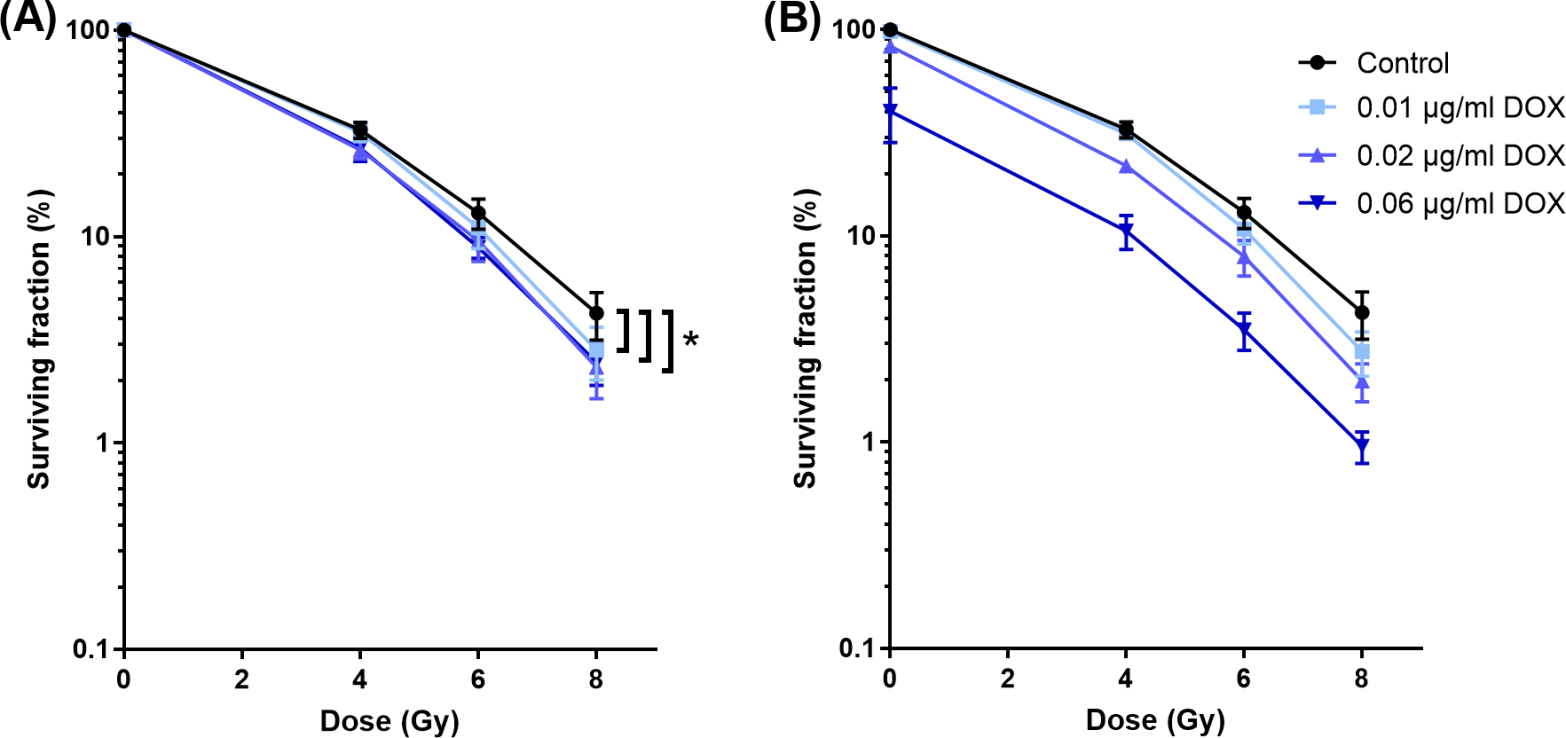
Cytotoxicity of DOX at different concentrations and RT. Surviving fraction as function of radiation dose for different DOX concentrations. Cells were exposed for 1 hour to DOX (0.01, 0.02 or 0.06 μg/mL) before RT. Surviving fraction calculated by samples treated with the corresponding DOX concentration (A) or by untreated sample (B). All DOX concentrations resulted in a significant enhancement of RT. In addition, 0.06 μg/ml DOX resulted in a larger RT effect compared to 0.01 μg/ml DOX (p<0.05).

The enhancement of the sensitivity to RT of cells was investigated for different DOX concentrations. Fig 2A shows the survival fraction of cells treated with DOX and RT relative to samples treated with equal chemotherapy treatment (equal DOX concentration and 0 Gy), whereas Fig 2B presents the surviving fraction relative to untreated samples (0 μg/ml DOX and 0 Gy). All DOX concentrations used in this experiment resulted in significant enhancement of the sensitivity to RT. In addition, DOX at a concentration of 0.06 μg/mL resulted in a small, but significant, increase in enhancement of the sensitivity to RT compared to 0.01 μg/mL (Fig 2A). DOX as a single treatment also showed, as expected, a concentration dependent increase of cell kill, which can be observed from the decreasing surviving fraction at 0 Gy for the different DOX concentration curves in Fig 2B.

### Effect of combinations of the modalities doxorubicin, HT and RT

Since HT not only causes drug release from TSL, but also sensitizes RT and DOX [27], the optimal sequence of the modalities in the concept of triggered radiosensitizer release was investigated. To avoid concentration differences of DOX due to the (incomplete) release of DOX from ThermoDox at 37°C, free DOX was used in these experiments. The DOX concentration was set to 0.02 μg/ml which led to a cell survival of approximately 10%, such that the effect of the combination treatments would be detectable. First, the effect of the sequence was investigated for the combination RT and HT and the combination RT and DOX.

### Radiotherapy and hyperthermia

**Fig 3:**
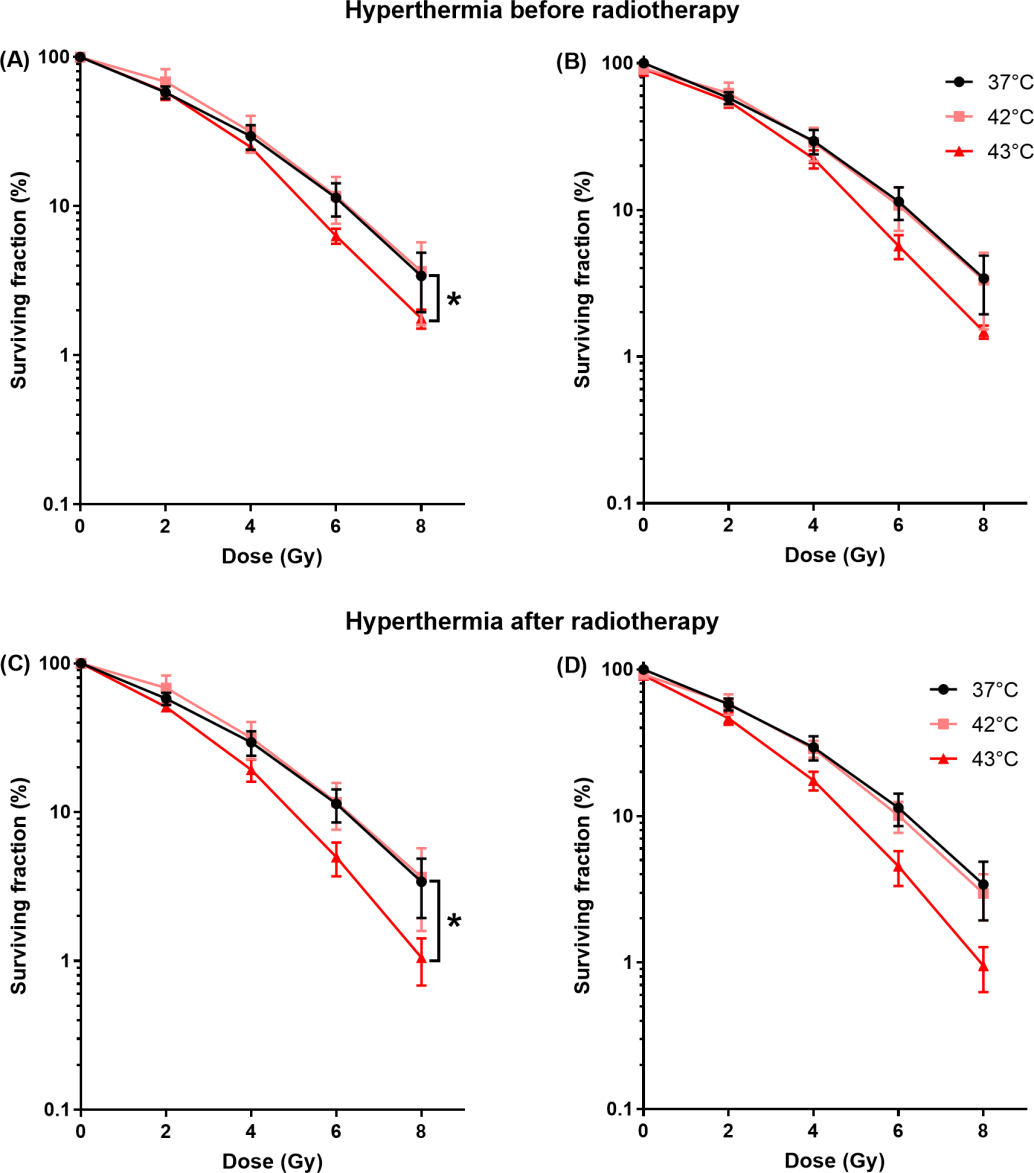
Cytotoxicity of hyperthermia at different temperatures before and after RT. Surviving fraction as function of radiation dose at different temperatures. Cells exposed for 1 hour to 37, 42 or 43°C before (A and B) or after (C and D) RT normalized for HT treatment at 0 Gy (A and C) and normalized for untreated samples (37°C and 0 Gy) (B and D). HT of 43°C resulted in significant enhancement of RT both before (A) and after RT (C). HT of 43°C resulted in the largest enhancement of the RT.

Fig 3 shows the surviving fraction as function of radiation dose of cells incubated at 37, 42 or 43°C for 1 hour before (A and B) or after (C and D) applying RT. Incubation of cells during 1 hour at 42°C did not improve the effect of RT on cell kill independent on the timing. HT at 42°C before or after RT resulted in a surviving fraction at 6 Gy (SF_6_) of 11.6% ± 4.1% and 11.7% ± 4.1%, respectively. In contrast, the effect of RT was significantly enhanced by incubating cells at 43°C. HT at 43°C applied before or after RT resulted in a SF_6_ of 6.3% ± 0.7% and of 5.0% ± 1.3%, respectively. HT at 43°C following RT resulted in a small, but significant larger enhancement of RT compared to HT at 43°C before RT. RT as a monotherapy resulted in a SF_6_ of 11.3% ± 2.8%.

### Radiotherapy and doxorubicin

**Fig 4:**
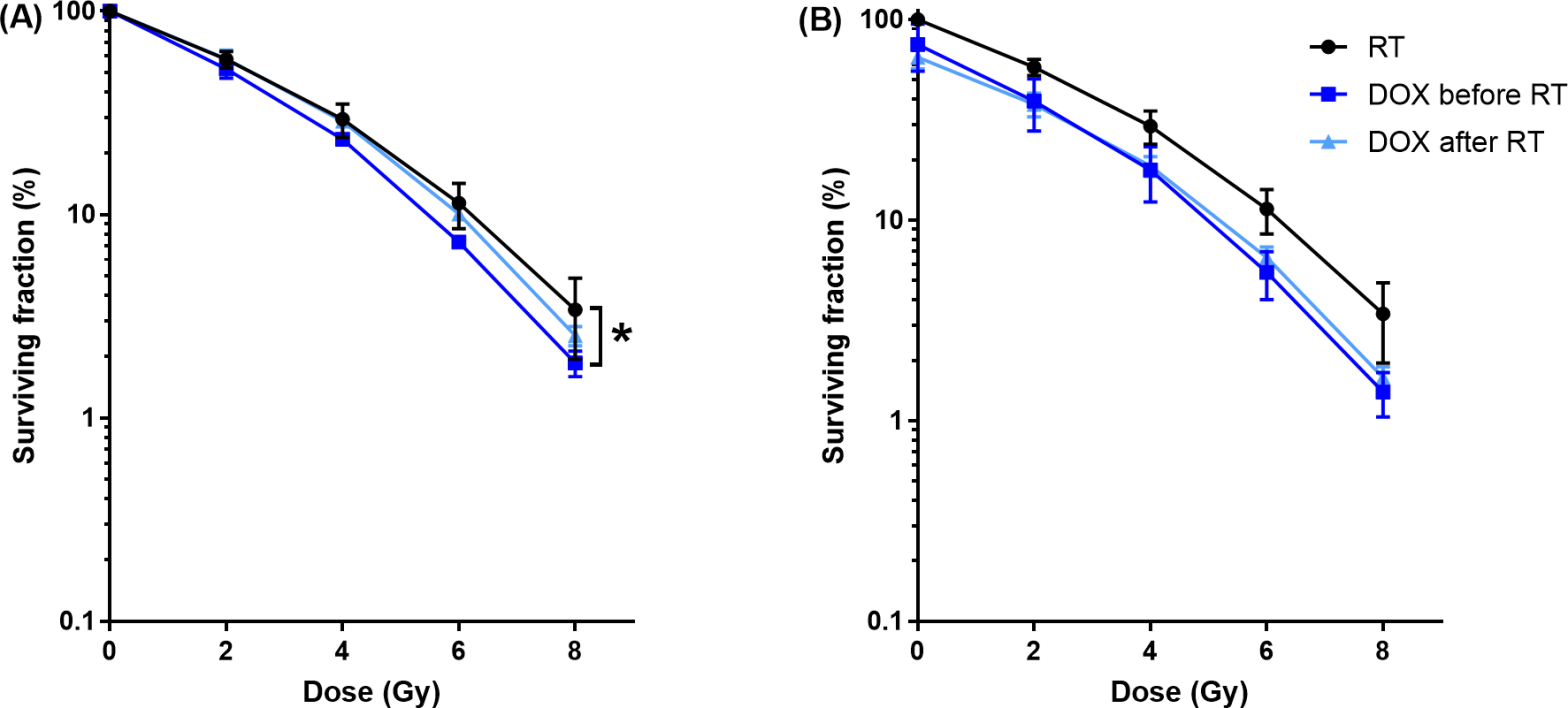
Cytotoxicity of DOX before or after RT. Surviving fraction as function of radiation dose. Cells were exposed for 1 hour to DOX (0.02 μg/mL) before or after RT, normalized for DOX at 0 Gy (A) and normalized for untreated samples (0 μg/ml and 0 Gy) (B). Only DOX before RT resulted in a significant enhancement of the RT.

Fig 4 shows the surviving fraction as function of radiation dose of cells exposed to DOX (0 or 0.02 μg/mL for 1 hour) before or after RT. Incubation of cells with DOX before RT resulted in a significant enhancement of the sensitivity to RT, however no enhancement of sensitivity was observed when DOX was applied after RT. The SF_6_ of DOX before and after RT were 7.32% ± 0.27% and 10.7% ± 0.71%, respectively.

### Doxorubicin and hyperthermia

**Fig 5:**
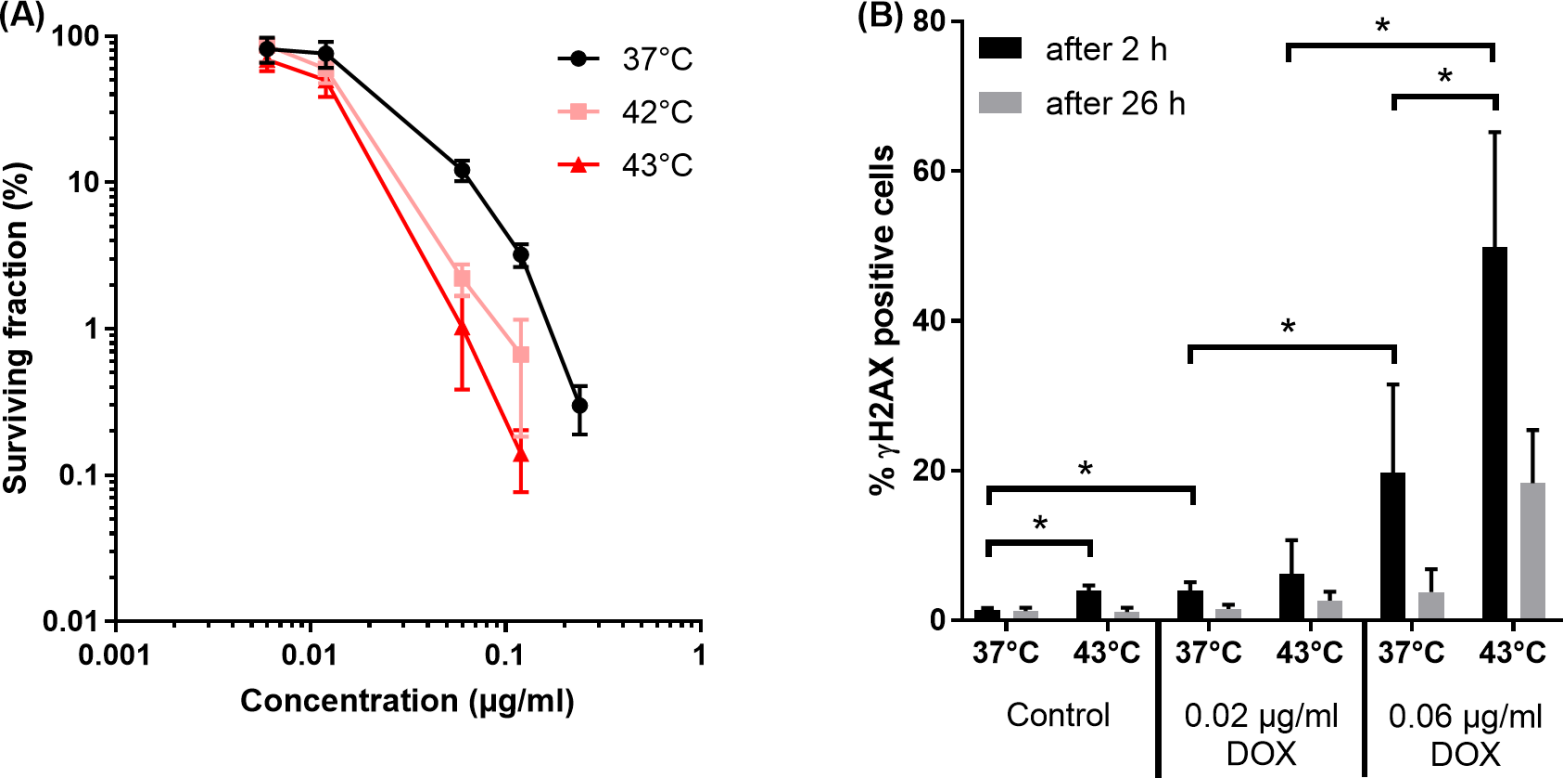
Cytotoxicity of hyperthermia at different temperatures and DOX. (A) Surviving fraction as function of DOX concentration for 1 hour at 37, 42 or 43°C. Cells were exposed for 1 hour to DOX at 37, 42 and 43°C. Both 42 and 43°C showed enhancement cytotoxicity of the DOX, which was the largest at 43°C. (B) Percentage γH2AX positive cells 2 and 26 hours after incubation with DOX (0, 0.02 or 0.06 μg/ml) for 1 hour at 37°C or 43°C. HT enhanced the percentage γH2AX positive cells both 2 and 26 hours after treatment.

Since in the proposed concept of triggered radiosensitizer release the primary role of HT is to trigger the release of the radiosensitizer, DOX and HT were always concurrently combined. The surviving fraction as function of DOX concentration at different temperatures is presented in Fig 5(A). HT enhanced the cytotoxicity of DOX, which was the largest at 43°C. At a surviving fraction of 1% the enhancement ratios of HT at 42°C and 43°C were 1.79 and 2.83, respectively. Note that HT as a single treatment resulted in limited toxicity, HT of 42 and 43°C resulted in surviving fractions of 96.73 ± 16.84% and 101.84 ± 0.21%, respectively.

These results were confirmed by measuring the percentage γH2AX positive cells 2 and 26 hours after incubated with DOX for 1 hour at 37 or 43°C (Fig 5B). An increase in DOX concentration resulted in an increase in percentage γH2AX positive cells both 2 and 26 hours after treatment, which was only significant 2 hours after DOX treatment. HT as a single treatment showed only 2 hours after treatment a significant enhancement of the percentage γH2AX positive cells, 26 hours after treatment the percentage γH2AX positive cells was similar to control cells. The combination DOX and HT resulted in enhanced percentage γH2AX positive cells compared to DOX as a single treatment, this was only significant at a DOX concentration of 0.06 μg/ml 2 hours after incubation. For all DOX and/or HT conditions 2 hours after treatment the percentage γH2AX positive cells were larger compared to 26 hours after treatment.

### Radiotherapy, doxorubicin and hyperthermia

**Fig 6:**
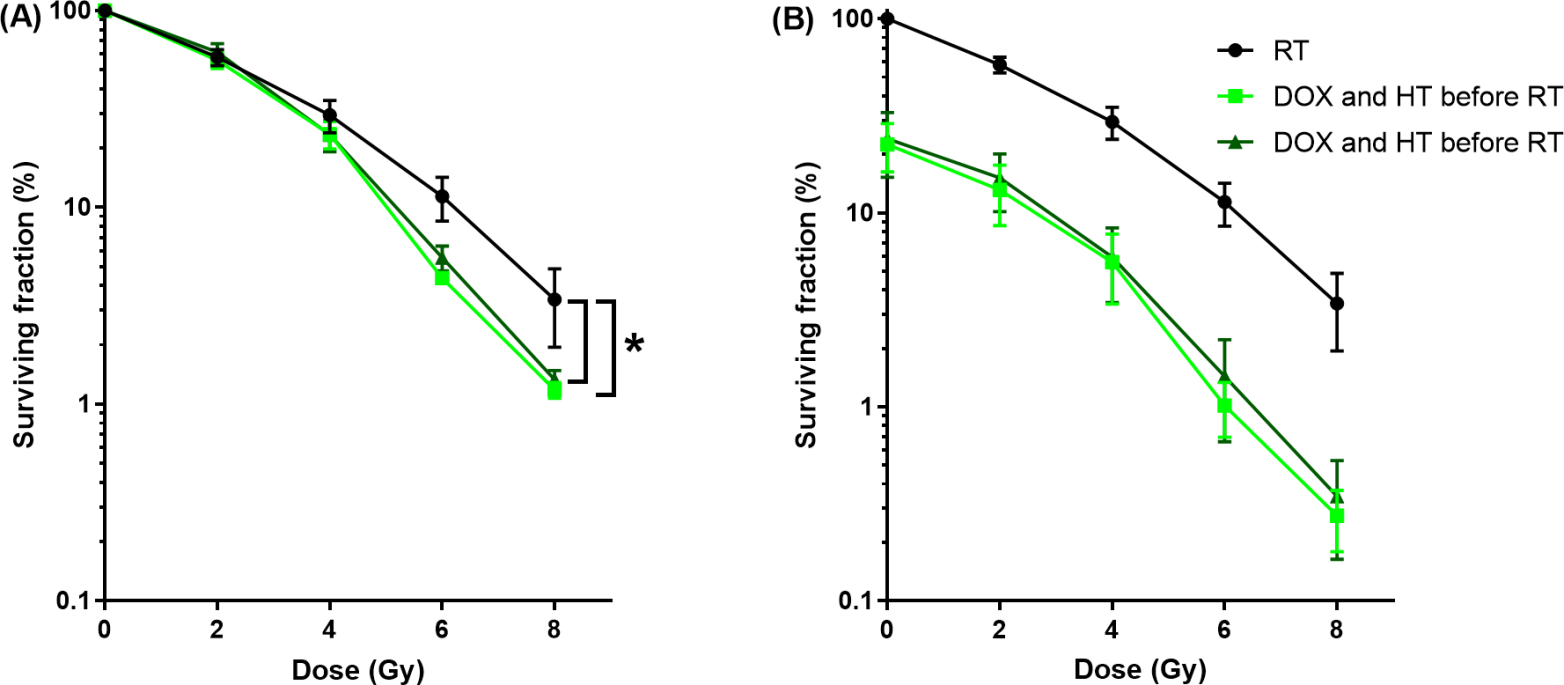
Cytotoxicity of HT and DOX before and after RT. Surviving fraction as function of radiation dose. Cells were exposed for 1 hour to DOX (0.02 μg/mL) at 43°C before or after RT. Both DOX and HT before and after RT resulted in a significant enhancement of the RT normalized for DOX and HT at 0 Gy (A) and normalized for untreated samples (0 μg/ml, 37°C and 0 Gy) (B).

Finally, the optimal sequence was investigated for all modalities in the proposed concept of triggered radiosensitzer delivery. In these experiments HT at 43°C was used, since HT at 42°C did not show enhancement of RT (Fig 3). Cells treated with DOX (0.02 μg/mL) at 43°C for 1 hour showed an equal and significant enhancement of the sensitivity to RT independent of the timing, i.e. before or after RT (Fig 6). The SF_6_ of DOX and HT before and after RT were 4.37% ± 0.15% and 5.56% ± 0.80%, respectively.

### Initial and prolonged double strand breaks after exposure to DOX, HT and RT

**Fig 7:**
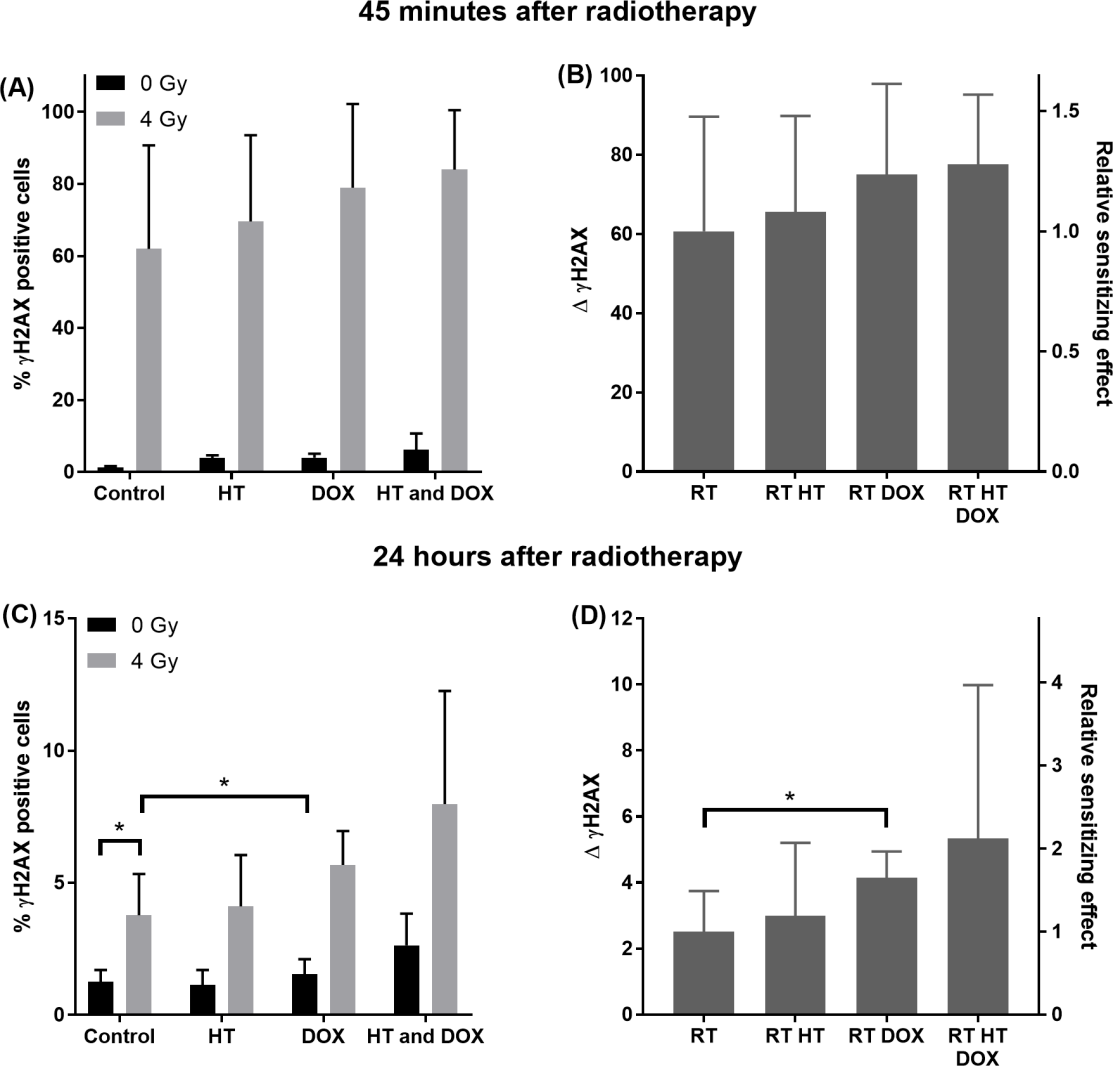
γH2AX staining after cells treated with DOX, HT and/or RT. Percentage γH2AX positive cells after incubated with DOX (0 or 0.02 μg/ml) for 1 hour at 37 or 43°C and irradiated at 0 or 4 Gy, 45 minutes (A and B) or 24 hours (C and D) after irradiation. Expressed in percentage γH2AX positive cells (A and C) and expressed in difference in percentage γH2AX cells of treatment with and without RT (0 and 4 Gy) and expressed in relative sensitizing effect (B and D).

The enhancement of RT by DOX and/or HT was further investigated by quantifying the percentage γH2AX positive cells 45 minutes and 24 hours after RT. Fig 7 shows the percentage γH2AX positive cells after treatment with DOX (0 or 0.02 μg/ml) at 37 or 43°C followed by RT (0 or 4 Gy). Flow cytometry measurements were performed either 45 minutes (A and B) or 24 hours (C and D) after RT. As a single treatment RT showed significant enhancement of the percentage γH2AX positive cells both 45 minutes and 24 hours after RT relative to control. To be able to assess the radiosensitizing effect of DOX and/or HT more clearly, the contribution of DOX and HT to the percentage of γH2AX positive cells in the absence of RT was subtracted from the percentage of γH2AX positive cells in the presence of RT, defining Δ γH2AX. Both HT, DOX and the combination HT and DOX in combination with RT resulted in an increase in Δ γH2AX, i.e. radiosensitizing effect, both 45 minutes and 24 hours after RT. This was only significant for the combination of DOX and RT 24 hours after irradiation.

## Discussion

To decrease toxicity of the radiosensitizers in normal tissue and increase the local efficacy of chemoradiation for local tumor control, we investigated *in vitro* the potential of the concept of triggered radiosensitizer release from TSL by heat followed by conventional radiotherapy. Triggered release of DOX from ThermoDox at 43°C resulted in comparable cell death as free DOX at 43°C, with and without RT. Exposure of cells to ThermoDox at 37°C alone or in combination with RT did not change the cell survival. DOX had a direct toxic effect on cells, and proved an effective enhancement of the RT which was only slightly concentration dependent. The sequence of the treatments in the concept triggered radiosensitizer release was important to achieve the largest enhancement of sensitivity to RT.

The concept of triggered radiosensitizer release was investigated by exposing cells to ThermoDox and DOX at 37 and 43°C followed by conventional RT. No toxicity was observed when cells were incubated with ThermoDox at 37°C, however ThermoDox at 43°C resulted in equal cytotoxicity compared to DOX at 43°C. In addition, ThermoDox at 37°C did not result in any radiosensitization. However, at 43°C, ThermoDox and DOX resulted in comparable radiosensitization. According to Li et al [33], 1 hour incubation of ThermoDox at 37°C results in 50% DOX release, which in this study would lead to a final DOX concentration of about 0.01 μg/mL. Exposure of HT1080 cells to a DOX concentration of 0.01 μg/mL did not result in cell death (Fig 4), therefore no toxicity was expected of ThermoDox at 37°C for 1 hour. In contrast, ThermoDox at 42°C results in 100% DOX release after 1 minute [33]. As a consequence, for almost the complete hour the DOX concentration of cells treated with ThermoDox is equal to cells treated with free DOX and therefore resulted in equal cytotoxicity. These results show the potential of the local radiosensitizer delivery concept for reducing toxicities in unheated normal tissue located in the beam path.

*In vivo* studies of ThermoDox combined with local HT show 4.7 to 26.7 times increase in DOX concentration in the tumor compared to treatment with free DOX, while leaving the concentration of DOX in normal tissue nearly unchanged [22–24]. To investigate the radiosensitization effect of this envisioned increased radiosensitizer concentration in the tumor, we investigated the concentration dependency of DOX based radiosensitization. Although all DOX concentrations used in this study (0.01 up to 0.06 μg/mL) resulted in an enhancement of the sensitivity to RT, a relatively large increase in DOX concentration was required to obtain a small increase in sensitivity to RT. To the best of the authors’ knowledge no data is available on the concentration dependence of the DOX radiosensitization. Other radiosensitizers, such as cisplatin [34] and docetaxel [35], show a larger concentration dependent radiosensitization compared to DOX. It could be expected that local triggered release of such radiosensitizers from TSL would result in a more pronounced concentration dependent radiosensitization effect.

Since HT not only causes drug release from TSL, but also sensitizes RT and chemotherapy, the optimization of the sequence of radiosensitizer, HT and RT is important to achieve the largest efficacy [26, 28, 29, 36]. In order to understand the contribution of each modality and maximize the effect of triggered radiosensitizer release, the effect of the sequence of DOX, HT and RT on radiosensitization was investigated. In this study temperatures of 42 and 43°C were investigated, since one hour incubation at these temperatures resulted in limited toxicity to the cells. Note that, compared to other cell lines, the HT1080 cells have been found to be relatively heat resistant [37, 38].

For the combination of RT and HT, a temperature dependent radiosensitization has been observed [37, 39, 40]. Here, HT at 43°C resulted in an enhanced sensitivity to RT, which was not observed at 42°C. As a result all subsequent HT experiments were performed at a temperature of 43°C. The largest sensitization was achieved when HT followed RT. The radiosensitization effect of HT is relatively small in HT1080 cells compared to other cell lines [28, 37, 40]. Both optimal temperature and optimal sequence of HT with respect to RT differs between cell lines [28, 37, 39, 40], however the underlying mechanism behind the optimal sequence of HT and RT is unknown. The main mechanism of HT mediated-radiosensitization is inhibition of the DNA damage repair proteins [29]. Therefore prolonged presence of DSB was expected after treatment of RT and HT compared to RT as a single treatment. However, in our study 24 hours after RT no enhancement of DSB was observed. This might be attributed to the recovery of the DNA damage repair protein function 6 hours after HT treatment as was shown by Van Oorschot [41]. In almost all cell lines, simultaneous HT and RT results in the largest sensitization effect [28], however simultaneous HT and RT is clinically impractical and therefore not investigated in this study.

Since the concept of local radiosensitizer release requires HT to release the radiosensitizer from the TSL, HT and DOX were always concurrently applied. HT potentiated the effect of DOX; at 42 and 43°C DOX showed consistently enhanced toxicity compared to 37°C. In addition, HT increased the DSB caused by DOX immediately and 24 hours after treatment. This enhanced toxicity is most likely caused by the increase in intracellular DOX uptake when cells were exposed to elevated temperatures [27, 42]. In the concept local triggered radiosensitizer delivery, the chemosensitization of HT could cause extra potentiation of tumor treatment.

In the combination of DOX and RT, only DOX treatment before RT resulted in an enhancement of the sensitivity to RT. This is most likely related to the radiosensitizing mechanism of DOX, as previously reported [43–45]. In addition, 24 hours after RT the amount of γH2AX positive cells was significantly enhanced by DOX compared to RT as a single therapy. Therefore we expect that the enhancement of RT by DOX is caused by an inhibition of the DSB repair, as was also observed by Bonner and Lawrence [44]. Possible mechanisms of inhibition of the DNA repair by DOX are stabilization of the DNA-topoisomerase II complex resulting in conformational changes of the DNA, cell kill in the radioresistant cell phase and free radical generation [44].

Finally, the combination of all treatment modalities for triggered radiosensitizer release was investigated. The sequences were limited to clinical relevant sequences for local radiosensitizer release. Since HT is required to release DOX from the TSL, HT and DOX were concurrently combined. In contrast, HT and RT were performed successively due to logistic limitations to be expected in a clinical setting. As a consequence, only the combinations of concurrent HT and DOX before or after RT were investigated. Concurrent HT and DOX before or after RT both resulted in equal enhancement of the sensitivity to RT. Since DOX before RT resulted in the largest enhancement of RT and HT after RT resulted in the largest enhancement of RT, it is plausible that HT and DOX concurrently before or after RT resulted in equal sensitization.

The primary goal of this study is to provide an in vitro proof of the triggered radiosensitizer delivery concept. We showed the potential of this concept in 1 cell line with 2 different radiobiological assays (i.e. clonogenic assay and γH2AX assay). To understand in more detail the mechanism underlying the sensitization effect of DOX, HT and RT, future experiments should be performed in multiple cell lines.

In the current *in vitro* study, HT can only act as a chemosensitizer and radiosensitizer at cellular level, by mechanisms such as inhibition of the DNA damage repair, enhanced DNA damage and by increasing the membrane fluidity. However, HT *in vivo* also sensitizes tissue to chemotherapy and RT at organ level, by mechanisms such as increasing blood flow, tissue oxygenation and increasing the vascular permeability [38]. The additional effects of HT *in vivo* compared to *in vitro*, further support our concept of local triggered radiosensitizer delivery *in vivo*.

Since ThermoDox is already commercially available for use in clinical trials, it was used in this study as an example of a radiosensitizer in a TSL (i.e. ThermoDox). Currently DOX is only occasionally used in the clinic as a radiosensitizer in the treatment of soft tissue sarcomas, hence a soft tissue sarcoma cell line (HT1080) was used in this study. In contrast, other radiosensitizers, which are also chemotherapeutic agents, exist which are more commonly used in the clinic and are already encapsulated in a TSL, including cisplatin [46], pyrimidine analogue gemcitabine (dFdC) [47] and 5-FU [48]. However, the objective of these studies is to decrease the toxicity of the chemotherapeutic agent by TSL encapsulation, and as a consequence have not been tested as a radiosensitizer for triggered local release in combination with RT. As mentioned, ThermoDox is rather leaky at 37°C, which leads to unnecessary release of radiosensitizer in normal tissue. Therefore a stable TSL containing the radiosensitizer should be prepared to avoid normal toxicity by the radiosensitizer.

## Conclusions

*In vitro* we showed enhancement of RT by the TSL only when the radiosensitizer was released by HT. These results demonstrated the potential of the local radiosensitizer release concept. Hyperthermia triggered local radiosensitizer delivery is able to decrease radiosensitizer-related toxicities in unheated normal tissue located in the beam path and at the same time enhance the efficacy of chemotherapy in heated tumor tissue. To maximize the effect of the individual treatments concurrent DOX and HT should be applied either before or after RT.

## Supporting information

S1 Fig Water bath and irradiation setup. A photo of the water bath (A) and irradiation setup (B). During incubation of the samples in the water bath and irradiation, well plates were wrapped with Parafilm.
(TIF)

S2 Fig Representative clonogenic assay images. Representative clonogenic assay images of untreated cells (A) and cells treated with radiotherapy 4 Gy (B), DOX 0.02 μg/ml (C) and HT 43°C (D) Only colonies that exists of at least 50 cells were counted (yellow dots). The plating efficiency was calculated from the number of colonies formed and the number of cells seeded according to Franken et al [30].
(TIF)

S3 Fig Representative flow cytometry results of γH2AX and PI fluorescence. Representative Flow Cytometry results of γH2AX and PI fluorescence of untreated cells and cells treated with hyperthermia (43°C), doxorubicin (0.02 μg/ml) and radiotherapy (4 Gy) 45 minutes (A) and 24 hours (B) after radiotherapy treatment. The percentage γH2AX positive cells was determined relative to the control sample. Since the fluorescence of PI highly depend on the concentration of PI, the width of the box to determine positive γH2AX labeled cells was adjusted to PI fluorescence.
(TIF)

S4 Fig Survival fraction as function of incubation time of at different temperatures. Cells exposed for 0 to 240 minutes to temperatures ranging between 41 and 45°C.
(TIF)
