# Supplementary figures and images for: Triggered radiosensitizer delivery using thermosensitive liposomes and hyperthermia improves efficacy of radiotherapy: An in vitro proof of concept study
Source: PLoS One. 2018 Sep 18;13(9):e0204063. doi: 10.1371/journal.pone.0204063 (PMC6143263; doi:10.1371/journal.pone.0204063)

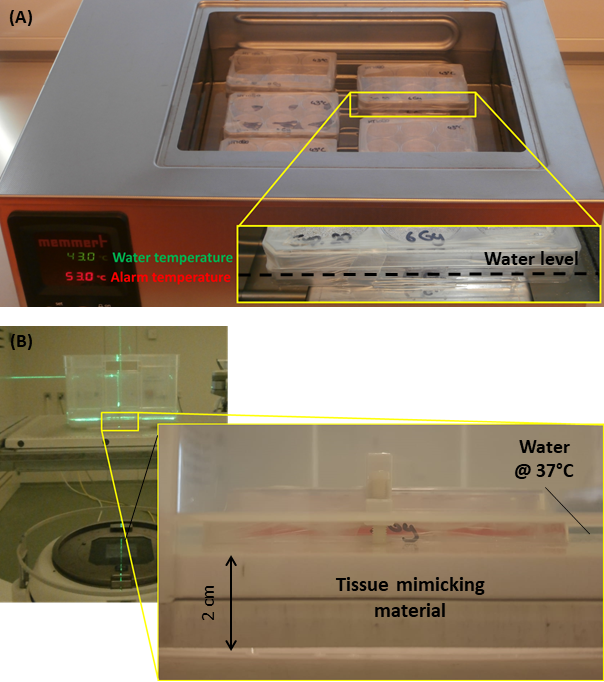

Supplement: S1 Fig — A photo of the water bath (A) and irradiation setup (B). During incubation of the samples in the water bath and irradiation, well plates were wrapped with Parafilm. (TIF) [file pone.0204063.s001.tif]

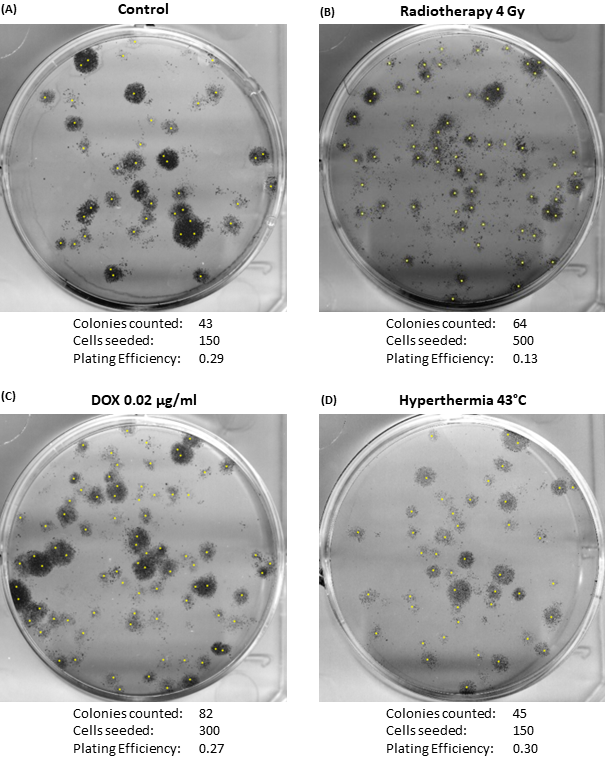

Supplement: S2 Fig — Representative clonogenic assay images of untreated cells (A) and cells treated with radiotherapy 4 Gy (B), DOX 0.02 μg/ml (C) and HT 43°C (D) Only colonies that exists of at least 50 cells were counted (yellow dots). The plating efficiency was calculated from the number of colonies formed and the number of cells seeded according to Franken et al [30]. (TIF) [file pone.0204063.s002.tif]

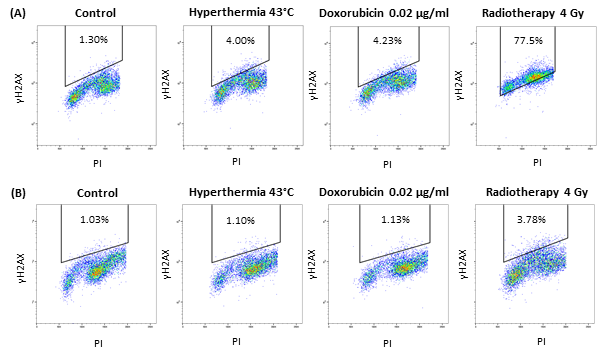

Supplement: S3 Fig — Representative Flow Cytometry results of γH2AX and PI fluorescence of untreated cells and cells treated with hyperthermia (43°C), doxorubicin (0.02 μg/ml) and radiotherapy (4 Gy) 45 minutes (A) and 24 hours (B) after radiotherapy treatment. The percentage γH2AX positive cells was determined relative to the control sample. Since the fluorescence of PI highly depend on the concentration of PI, the width of the box to determine positive γH2AX labeled cells was adjusted to PI fluorescence. (TIF) [file pone.0204063.s003.tif]

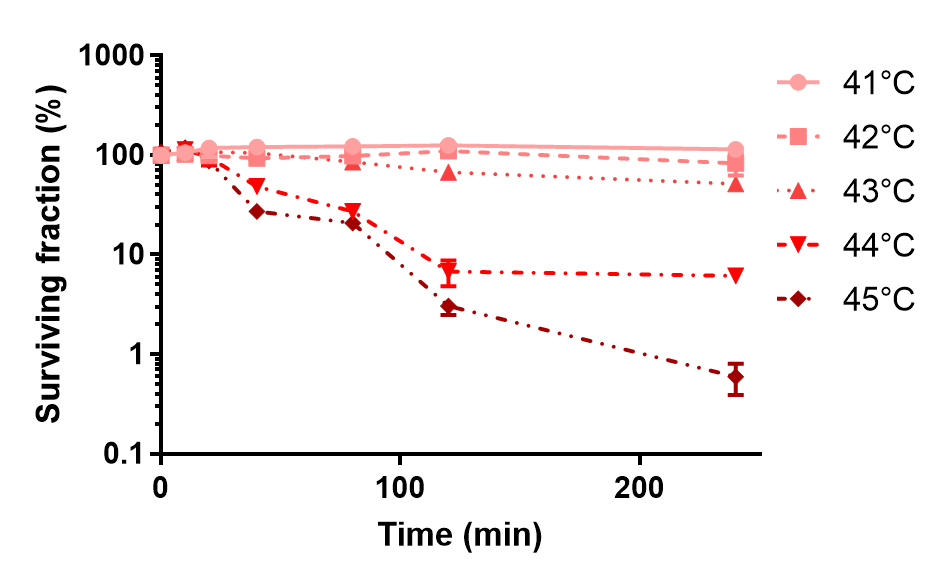

Supplement: S4 Fig — Cells exposed for 0 to 240 minutes to temperatures ranging between 41 and 45°C. (TIF) [file pone.0204063.s004.tif]
